# Interventions for Strengthening General Self-Efficacy Beliefs in College Students: An Integrative Review

**DOI:** 10.1590/0034-7167-2023-0192

**Published:** 2023-12-08

**Authors:** Lais Palotta Balderrama Gueroni, Daniele Alcalá Pompeo, Letícia Palota Eid, Marcos Antonio Ferreira, Carlos Alberto da Cruz Sequeira, Luciano Garcia Lourenção

**Affiliations:** I Faculdade de Medicina de São José do Rio Preto. São José do Rio Preto, São Paulo, Brazil; II Universidade Federal de Jataí. Jataí, Goiás, Brazil; III Universidade Federal de Mato Grosso do Sul. Campo Grande, Mato Grosso do Sul, Brazil; IV Escola Superior de Enfermagem do Porto. Porto, Portugal; V Universidade Federal do Rio Grande. Rio Grande, Rio Grande do Sul, Brazil

**Keywords:** Self Efficacy, Review, Health Promotion, Student Health, Students, Autoeficacia, Revisión, Promoción de la Salud, Salud del Estudiante, Estudiantes, Autoeficácia, Revisão, Promoção da Saúde, Saúde do Estudante, Estudantes

## Abstract

**Objective:**

To assess the evidence regarding the effectiveness of interventions aimed at strengthening self-efficacy beliefs in college students.

**Methods:**

Integrative Review conducted on the Lilacs, PubMed, CinahL, Cochrane Collaboration Databases, Scopus, and PsycInfo databases. The methodological quality of the studies was assessed using tools proposed by the Joanna Briggs Institute, and the results were analyzed descriptively.

**Results:**

Out of the 10 selected studies, six demonstrated that interventions aimed at strengthening self-efficacy were effective (Levels of Evidence II and III), and four revealed contrary results (Levels of Evidence I and II). Programs aimed at enhancing self-efficacy should include content on positive mental health, psychoeducation strategies, cover a period of eight to twelve weeks, and consider the completion of homework assignments.

**Conclusion:**

The synthesis of evidence pointed to pathways for building an effective self-efficacy strengthening program to be implemented in universities.

## RESUMO

**Objetivo:**

Avaliar as evidências sobre a efetividade das intervenções para fortalecer as crenças de autoeficácia em universitários.

**Métodos:**

Revisão Integrativa, realizada nas bases de dados *Lilacs, PubMed, CinahL, Cochrane Collaboration Databases, Scopus* e *PsycInfo*. A qualidade metodológica dos estudos foi avaliada por meio de ferramentas propostas pelo *Joanna Briggs Institute* e os resultados foram analisados de forma descritiva.

**Resultados:**

Dos 10 estudos selecionados, seis demonstraram que as intervenções para fortalecer a autoeficácia foram efetivas (níveis de evidências II e III) e quatro revelaram resultados contrários (níveis de evidências I e II). Os programas direcionados ao fortalecimento da autoeficácia devem possuir conteúdo sobre saúde mental positiva, estratégias de psicoeducação, abranger o período de oito a doze semanas e considerar a realização de exercícios para casa.

**Conclusão:**

A síntese das evidências apontou caminhos para a construção de um programa efetivo de fortalecimento das crenças de autoeficácia a ser implementado em universidades.

## RESUMEN

**Objetivo:**

Evaluar las evidencias sobre la efectividad de las intervenciones para fortalecer las creencias de autoeficacia en estudiantes universitarios.

**Métodos:**

Revisión Integrativa realizada en las bases de datos Lilacs, PubMed, CinahL, Cochrane Collaboration Databases, Scopus y PsycInfo. La calidad metodológica de los estudios se evaluó utilizando herramientas propuestas por el Instituto Joanna Briggs y los resultados se analizaron de forma descriptiva.

**Resultados:**

De los 10 estudios seleccionados, seis demostraron que las intervenciones para fortalecer la autoeficacia fueron efectivas (niveles de evidencia II y III) y cuatro arrojaron resultados contrarios (niveles de evidencia I y II). Los programas dirigidos al fortalecimiento de la autoeficacia deben incluir contenido sobre salud mental positiva, estrategias de psicoeducación, abarcar un período de ocho a doce semanas y considerar la realización de ejercicios para hacer en casa.

**Conclusión:**

La síntesis de evidencia señala el camino para la construcción de un programa efectivo de fortalecimiento de las creencias de autoeficacia que debe implementarse en las universidades.

## INTRODUCTION

University education represents an important stage in cognitive development and poses numerous challenges for students transitioning from adolescence to young adulthood. Scientific literature provides substantial insights into psychological stress and the significant rise in mental health issues within this population, primarily stemming from the necessity to adapt to the new demands of higher education. These demands include residing in a distant location from their place of origin, limited resources, and a competitive environment^(1)^. This condition is linked to elevated stress levels^(2)^, anxiety, depression^(3-4)^, reduced quality of life^(5)^, profound emotional distress, and even instances of suicide^(6-7)^ among university students.

Given this alarming backdrop, further exacerbated by the covid-19 pandemic^(8)^, there is a pressing need for interventions targeting the mental health of this specific demographic to bolster their positive emotional resources, especially at the outset of their academic journey^(3)^. Promoting mental health within the realm of primary prevention, before the onset of intense stress and morbidity, is considered a promising approach for enhancing individual qualities and optimizing their potential.

The pillars of mental health encompass factors that assist individuals in integrating and adapting to the world while affording protection against mental illness. Notable examples include personal satisfaction, a pro-social attitude, self-control, autonomy, problem-solving/achievement, and interpersonal relationship skills^(9)^.

Self-efficacy is recognized as a fundamental skill for sustaining the mental health of young university students^(9)^ and is embedded in the context of personal achievement. It is defined as an individual’s belief in their own competence and ability to execute and organize tasks to achieve the desired outcomes^(10)^. These beliefs shape how individuals engage in pursuing their goals, their perseverance in the face of adversity and setbacks, their utilization of resilience, their willingness to confront challenges, and also serve as significant indicators of stress levels, anxiety, depression, personal accomplishment^(11)^, and academic success^(12-13)^.

Individuals with a heightened sense of self-efficacy typically gravitate toward more demanding tasks, invest greater effort, maintain focus on their intended objectives, exhibit greater persistence, visualize success, and perceive problems as opportunities. Conversely, individuals with low self-efficacy tend to construct negative anticipatory scenarios, view themselves as ineffective or incapable, and cultivate mental images of failure^(11)^.

Therefore, considering the elevated and escalating prevalence of mental health issues among university students and the potential for academic and professional success, universities must effectively intervene to address this concerning scenario. Urgently needed are interventions that reinforce protective psychological resources, mitigate stress, and prevent adverse outcomes or the onset of mental health disorders. Self-efficacy is a vital resource that should be cultivated, particularly during the educational journey.

Scientific literature indicates interventions aimed at enhancing the mental health of undergraduate students have yielded promising results, yet they have not adequately addressed the gaps within this research domain. These gaps include questions such as: Which emotional variables should be addressed? How should they be approached? What is the requisite timeframe for developing a skill that positively impacts mental health outcomes? What are the individual and academic characteristics that necessitate the adoption of in-person, remote, or hybrid methods?

In light of the foregoing and the absence of protocols and systematic review reports registered in the International Prospective Register of Systematic Reviews (PROSPERO), there is a compelling need to engage in discussions within the scientific literature regarding interventions capable of promoting, enhancing, or strengthening the general self-efficacy beliefs of university students.

## OBJECTIVE

To evaluate the scientific evidence regarding the effectiveness of interventions for promoting and/or strengthening general self-efficacy beliefs in university students.

## METHODS

### Ethical Considerations

This study adhered to the ethical standards established by national and international regulatory bodies. The ideas of the authors of the publications used were duly credited and respected.

### Design

This constitutes an integrative literature review (IR), conducted through six stages: Identification of the theme and formulation of the research question; Literature search and sampling; Categorization of studies; Evaluation of studies; Interpretation of results, and Synthesis of knowledge^(14)^. It was carried out based on an IR protocol developed specifically for this study.

### Formulation of the Research Question

The guiding question was formulated based on the PICOS acronym to achieve greater sensitivity in retrieving studies that would answer the research question: P (patients/context): university students; I (intervention): strategies for strengthening/promoting general self-efficacy beliefs; C (comparison): other strategies or without comparison; O (outcome): levels of general self-efficacy perception; S (study type): all methodological designs, with an emphasis on experimental or quasi-experimental studies. Thus, the following guiding question was formulated: “What are the interventions for promoting and/or strengthening general self-efficacy beliefs in university students?”

### Literature Search and Sampling

The search for studies was conducted by three researchers (P1, P2, P3) with expertise in data retrieval and mental health promotion, independently and simultaneously in the following databases: Latin American and Caribbean Literature in Health Sciences (Lilacs), PubMed, Cumulative Index to Nursing & Allied Health Literature (CinahL), Cochrane Collaboration Databases, Scopus, and PsycInfo. There was no time limit for selecting studies, with data collection from manuscripts published up to July 31, 2022. Disagreements were resolved through a consensus meeting, comparing the results of the searches and verifying the differences in findings.

For the development of the search strategy, controlled MeSH (Medical Subject Headings) descriptors and their respective synonyms (entry terms) were selected as follows:

i) #1 Self-efficacy (Efficacy, Self); ii) #2 Health promotion (Promotion, Health OR Promotions, Health OR Promotion of Health OR Health Promotions OR Promotional Items OR Item, Promotional OR Items, Promotional OR Promotional Item OR Wellness Programs OR Program, Wellness OR Programs, Wellness OR Wellness Program OR Health Campaigns OR Campaign, Health OR Campaigns, Health OR Health Campaign); iii) #3 Mental Health (Health, Mental OR Mental Hygiene OR Hygiene, Mental); iv) #4 Students (Student OR School Enrollment OR Enrollment, School OR Enrollments, School OR School Enrollments); v) #5 Student health services (Health Services, Student OR Health Service, Student OR Service, Student Health OR Student Health Service OR Services, Student Health OR Health Services, University OR Health Service, University OR Service, University Health OR University Health Service OR University Health Services OR Services, University Health).

The MeSH term combinations in the databases were combined using the Boolean operator AND, and the entry terms using the Boolean connector OR. Three combinations were adopted: a) #1 AND #2 AND #3; b) #1 AND #2 AND #4; c) #1 AND #5.

The established inclusion criteria were as follows: i) Investigations addressing interventions for promoting and/or strengthening self-efficacy in university students, ii) Original articles, systematic reviews, and meta-analyses, iii) Articles in Portuguese, English, and Spanish. No specific time frame for publication was set. Exclusion criteria included: i) Editorials and studies from books, theses, and dissertations that were not in the format of scientific articles, as well as non-systematic reviews, ii) Investigations focusing on self-efficacy related to a procedural task or specific skill, such as administering medication injections and improving clinical consultation skills.

The study selection process was independently conducted by researchers P1 and P2. Initially, titles and abstracts were reviewed, and a relevance test was applied, consisting of inclusion and exclusion criteria. Duplicate articles in the databases were counted only once. Subsequently, selected studies were read in their entirety, and those that did not meet the eligibility criteria were excluded. Any discrepancies in selection between the two researchers (P1 and P2) were discussed and analyzed by researcher P3 to reach a consensus.

**Figure 1:**
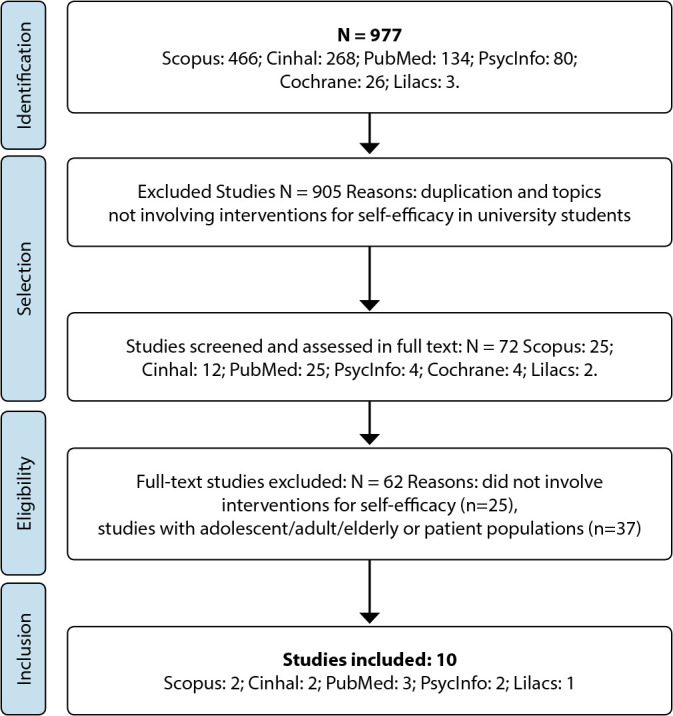
Flowchart of study identification, selection, eligibility, and inclusion in the integrative review, 2022

Figure 1 illustrates the process followed for study inclusion in the integrative review, using the flowchart proposed by PRISMA (Preferred Reporting Items for Systematic Reviews and Meta-Analyses)^(15)^.

### Categorization and Evaluation of Studies

Data extraction was independently performed by researchers P1 and P2 using an instrument specifically developed for this study. It included the following information: article identification (title, journal, publication year, study location), research purpose, methodological characteristics (design, number of participants, rigor, biases), results (intervention proposal, number of sessions, duration, strategy employed, involved professional category, effectiveness), level of evidence (LE), and article quality. Researcher P3 assessed the accuracy of the collected data and assisted in resolving discrepancies.

The level of evidence was determined following Melnyk and Fineout’s hierarchical model^(16)^. The quality of the included articles was assessed using Joanna Briggs Institute (JBI) tools: Checklist for Quasi-Experimental Studies (nine items), Checklist for Randomized Controlled Trials (13 items), and Checklist for Systematic Reviews (11 items). These checklists evaluate data reliability by examining methodological quality and potential biases in study design and execution. Each assessed item had four possible responses: yes, no, uncertain, or not applicable^(17)^. As a result, studies were categorized as follows: low risk of bias (≥70% of yes responses), moderate risk of bias (50% to 69% of yes responses), and high risk of bias (≤49% of yes responses).

Primary study information needed to address the review’s objective was presented in summary tables, and the results were analyzed descriptively.

## RESULTS

Ten studies on interventions for promoting and/or strengthening general self-efficacy beliefs in university students were selected. Out of these, one was published in 2016, one in 2017, one in 2018, three in 2019, three in 2020, and one in 2021. The studies were conducted in South America (n=2), North America (n=2), Europe (n=2), Asia (n=3), and Oceania (n=1).

Among the included studies, four did not yield favorable results regarding the promotion of self-efficacy after some form of intervention: one systematic review (LE: I) and three randomized clinical trials (LE: II). The remaining six studies showed favorable results (five studies with LE: III and one with LE: II). The assessment of methodological quality and potential biases in the design and conduct of the studies revealed moderate and low bias risks. The main results of the primary studies are presented in Charts 1 and 2.

**Chart 1:** Studies that achieved effective results in promoting and/or strengthening the general self-efficacy of university students, 2022 (n=6)

Study	Objective	Intervention	Strategy/Content Covered	Quality
Brennan et al., 2016^(18)^ USA	Test a stress control and relaxation program in medical students.	Single group=42 In-person intervention conducted in 8 sessions by a physician and psychologist.	Strategy/Content Covered Psychoeducation on positive mental health. Behavioral strategies: muscle relaxation, breathing exercises, meditation, guided imagery. Homework exercises: yes.	LE: III JBI: 66,6% (MRB)
Guo et al, 2017^(13)^ China	Test a positive psychotherapy program in nursing students.	Intervention group=34 and Control group=42 In-person intervention conducted in subgroups of 10 to 12 participants, with 8 sessions of 1.5 hours per week, by a nurse.	Psychoeducation on positive mental health. Homework exercises: yes.	LE: II JBI: 69,2% (MRB)
Terp et al., 2019^(19)^ Sweden	Test a stress management program in nursing students.	Intervention group=117 and Control group=44. In-person intervention conducted in 10 sessions of 2 hours per week, by a psychologist.	Cognitive-behavioral therapy and psychoeducation. Homework exercises: yes.	LE III JBI: 88,8% (LRB)
Brett et al., 2020^(20)^ USA	Test a health promotion program in university students.	Single group=45. Online intervention, with 12 sessions, one access per week, by a psychologist.	Health education/psychoeducation. Homework exercises: yes.	LE III JBI: 66,6% (MRB)
Ribeiro et al., 2020^(21)^ Brazil	Test a self-esteem strengthening program in nursing students.	Single group=74. Online intervention, with 10 sessions of 1 hour and 30 minutes, once a week, by a nurse.	Psychoeducation and persuasion. Homework exercises: yes.	LE III JBI: 55,5% (MRB)
Severian et al., 2021^(22)^ Brazil	Test a self-efficacy and self-esteem strengthening program in nursing students.	Single group=90. In-person intervention, with 10 sessions of 40 minutes each, once a week, by a nurse.	Psychoeducation and persuasion. Homework exercises: yes.	LE III JBI: 66,6% (MRB)

*Notes: LE: level of evidence; JBI: Joanna Briggs Institute; LRB: low risk of bias; MRB: moderate risk of bias.*

**Chart 2:** Studies that did not achieve effective results in promoting and/or strengthening the general self-efficacy of university students, 2022 (n=4)

Study	Objective	Intervention	Strategy/Content Covered	Quality
Li et al., 2018^(23)^ China	Review the literature on mental health promotion interventions in nursing students.	Different proposals, with varying durations and formats, conducted by a healthcare team.	Psychotherapies and behavioral strategies (mindfulness, music therapy, and physical exercises), psychoeducation (stress reduction and positive mental health).	LE: I JBI: 90,9% (LRB)
Recabarren et al., 2019^(24)^ Sweden	Test a stress management program in university students.	Intervention group=31 and Control group=32. In-person intervention conducted in groups of 8 students, with 8 sessions of 2 hours per week, by a psychologist.	Cognitive-behavioral therapy and behavioral strategies (mindfulness and skills training). Homework exercises: yes.	LE: II JBI: 69,2% (MRB)
Farrer et al., 2019^(25)^ Australia	Test a mental health promotion program in university students	Intervention group=102 and Control group=98. Online intervention, over a period of 6 weeks, led by a psychologist.	Cognitive-behavioral therapy, psychoeducation, behavioral strategies (mindfulness, mindfulness, and skills training). Homework exercises: yes.	NE: II JBI: 76,9% (LRB)
Yang et al., 2020^(26)^ China	. Test a program to improve health behaviors in university students.	Intervention group=263 and Control group=269. In-person intervention, conducted in 8 sessions of 2 hours per week, by a health instructor.	Health education (nutrition, sleep, physical exercises, stress reduction, and internet addiction prevention).	LE: II JBI: 61,5% (LRB)

*Notes: LE: level of evidence; JBI: Joanna Briggs Institute; LRB: low risk of bias; MRB: moderate risk of bias.*

Programs for promoting self-efficacy were mostly conducted over a period of eight to ten weeks, in groups of varying sizes (eight to 74 students), led by psychologists and nurses, in both face-to-face and online formats, using psychoeducation strategies, socio-emotional skills training, and cognitive-behavioral therapy. In psychoeducation, the most frequently addressed topics included personal and university routine organization, positive mental health, nutrition, and stress reduction tools. The interventions aimed at skills training were relaxation techniques, breathing exercises, meditation, guided imagery, mindfulness, music therapy, and physical exercises. All examined programs emphasized the presence of homework, meaning exercises for the students to perform or reflect upon after the intervention sessions (Charts 1 and 2).

## DISCUSSION

The results of this integrative review demonstrate that interventions targeting the mental health of university students can enhance their health behaviors, including their general self-efficacy levels. A limited body of work related to this field of knowledge was identified, particularly in Brazil, indicating a significant gap that needs consideration. Strong scientific evidence has shown that holding more optimistic personal beliefs about events influencing one’s life is positively correlated with improved learning, confidence, responsibility, self-esteem, and overall well-being^(12)^. Moreover, it helps mitigate the harmful effects stemming from stress^(19,27)^ and symptoms of anxiety and depression^(13,19)^.

The synthesis of evidence from this IR offers insights for the development of a program aimed at strengthening general self-efficacy beliefs to be implemented in universities. Among the studies evaluated, six presented interventions that had a positive impact on students’ perception of personal efficacy. However, four investigations concluded that the interventions did not influence participants’ self-efficacy.

It was noted that none of the studies considered self-efficacy as a fundamental construct in their intervention proposals. The analyzed programs were mostly mixed, characterized by multidimensional and integrated features. Such interventions are frequently encountered^(28)^, explained by the comprehensive framework of mental health, which encompasses various aspects, with self-efficacy being one of them. Most of the time, this broad landscape makes it challenging to determine whether the actual mechanism that led to the enhancement of participants’ mental well-being was singular or multifactorial, complicating evidence-based approaches.

For instance, the study with LE I incorporated psychotherapies, mindfulness, music therapy, and stress control strategies, resulting in positive outcomes for anxiety and stress but negative outcomes for self-efficacy^(23)^. Consequently, some caution is warranted when interpreting the results due to disparities in the duration and format of the interventions analyzed, as well as variations in their scope and objectives.

Similarly, the study with LE II, which examined a stress reduction program, achieved better outcomes in terms of anxiety control, psychological symptoms, interpersonal relationships, sense of coherence, self-compassion, and quality of life. However, it did not significantly affect social anxiety, support, and self-efficacy^(24)^. One possible explanation is that the intervention primarily targeted stress, employing numerous techniques and strategies focused on this aspect. Building self-efficacy is rooted in positive mental health, aiming to instill in individuals the belief in their ability to perform specific tasks, a facet not addressed in the study’s proposed sessions.

Another LE II investigation, focused on health promotion, did not yield significant results in terms of anxiety, depression, psychological distress, and self-efficacy. Nevertheless, it was effective in improving social anxiety and academic self-efficacy. These outcomes may be attributed to the limited duration of the intervention, which spanned only six weeks, the participants’ conditions (some of whom already had severe depression and bipolar disorder), and the intervention’s theme, which centered on coping with these conditions^(25)^.

An Asian study with LE II assessed an intervention designed to enhance the health behaviors of university students through health education. While participants demonstrated improved health behaviors, the educational program did not have a notable impact on the students’ well-being and self-efficacy^(26)^. This may be due to the content offered, which was related to nutrition, sleep, physical exercise, stress reduction, and Internet addiction prevention, with minimal or no emphasis on strengthening self-efficacy beliefs.

Six randomized and non-randomized clinical trials (LE II and III) underscored the strengthening of self-efficacy beliefs following interventions^(13,18-22)^. These studies shared certain common characteristics, such as a thematic focus on positive mental health through psychoeducation, intervention periods ranging from eight to 12 weeks, and the inclusion of homework exercises. The number of participants and the mode of delivery, whether in-person or online, did not significantly affect the programs’ effectiveness.

The interventions that primarily focused on positive mental health were possibly more effective because this construct is centered on human well-being. This ideal state of functioning is based on attributes such as personal satisfaction, a pro-social attitude, self-control, interpersonal relationships, problem-solving, self-realization, and autonomy^(9)^. The focus of positive mental health is to promote an individual’s qualities in optimizing their potential^(9)^, which includes self-efficacy.

Consistent with these findings, a study involving 73 American university students in the health field demonstrated that participants with higher scores in positive mental health reported higher levels of self-efficacy and greater commitment to their studies. Furthermore, the authors suggested that the constructs of positive mental health, self-efficacy, and academic success appear to be interconnected. Each construct can influence the others, triggering a cascade of effects on student success^(29)^.

A study involving 2,160 university students identified that daily stress is associated with the presence of anxiety, depression, and reduced subjective well-being. However, the authors recommended that efforts to promote mental health should not solely target stress reduction, as self-efficacy acted as a mediator in predicting positive or negative mental health. Thus, the study provided evidence that self-efficacy is an important target for psychological interventions to better protect students’ mental health^(30)^.

Psychoeducational-based interventions have the potential for broad reach, as they can be implemented by any knowledgeable professional to provide accurate information on health issues and enable effective self-management. Multiple pieces of evidence have indicated the efficacy of psychoeducational interventions in mental health^(31-32)^, highlighting the central role of nurses in planning and executing programs with this approach^(32)^.

Recently, a systematic review aimed to compile evidence on psychoeducational interventions in the context of depression among adolescents. The authors concluded that these programs encompassed a wide variety of approaches, whether individual or group-based, as well as diverse settings (community, school, service) and modes of communication (print, online, game, lecture). Consequently, it was determined that these actions can take on multiple formats and approaches, including integration into a single program^(28)^.

Similarly, another review showed that psychoeducational programs are conducted by a variety of professionals, including nurses, psychiatrists, psychologists, or other healthcare workers^(33)^. However, the evidence suggested that the success of a program is closely related to its execution, including facilitator motivation, skills, and the therapeutic relationship^(28,33)^.

Additionally, it was found that the presence of homework assignments after each session was effective in all studies with positive outcomes for self-efficacy. Thus, it is posited that these extra-session exercises can promote greater engagement and a sense of belonging among participants.

Regarding the duration of the intervention, the evidence ranged from eight to twelve weeks, suggesting that this initial period may be potentially pivotal in generating or provoking behavioral changes. This finding aligns with data compiled in a review of clinical studies, which recommended that the number of psychotherapeutic sessions can vary from 5 to 12 weeks, each lasting 45 to 60 minutes^(33)^.

Concerning the sustainability of the beneficial effects of mental health interventions, although there are significant gaps, relevant data lead us to believe in the need for the maintenance and consistency of preventive programs. Recent evidence from a meta-analysis suggested longer-term effects of interventions aimed at reducing anxiety (7 to 12 months) and depressive symptoms (13 to 18 months), with shorter durations in the case of interventions targeting positive mental health promotion and stress reduction (3 to 6 months)^(34)^.

Based on the literature analyzed, this integrative review recommends an intervention program for university students without established mental disorders, consisting of weekly sessions, ten sessions lasting one to one and a half hours each, and focusing on positive mental health. The evidence obtained indicated that key topics for a potentially effective intervention to strengthen general self-efficacy beliefs can encompass: 1) Self-awareness and self-concept; 2) Stress, anxiety, and frustration; 3) Coping mechanisms and resilience; 4) Healthy lifestyle habits (sleep, nutrition, and physical activity); 5) Organizing university routines and goal planning; 6) Interpersonal relationship skills and flexibility; 7) Strengthening personal beliefs; 8) Self-confidence and personal security; 9) Decision-making skills; 10) Self-management and self-advocacy. Fundamental strategies include psychoeducation in its various forms and approaches and the inclusion of homework assignments at the end of each session. Furthermore, it is recommended that interventions commence early, in the first year of higher education, and are reinforced throughout the undergraduate period.

### Limitations of the Study

This study had certain limitations that merit consideration. Most of the included studies gathered data solely from immediate post-tests, potentially limiting our understanding of the temporal effectiveness of the interventions. Another significant limitation was the limited number of studies available, which hindered an investigation into the most effective approaches for different course profiles. Among the analyzed studies, one involved medical students, four involved nursing students, and five encompassed students from various courses.

### Contributions to the Field of Nursing and Public Health

The findings of this Integrative Review (IR) suggest that the inclusion of programs designed to bolster self-efficacy beliefs within university settings can contribute to the enhancement of students’ health behaviors and the prevention of mental health disorders. Current programs tend to target students who are already experiencing some form of mental disorder. Consequently, this study represents a step forward in the context of primary prevention by harnessing positive resources to prevent negative mental health conditions. It initiates this process with self-efficacy beliefs, which have proven to be amenable to modification through brief interventions and exhibit a buffering effect against common symptoms of stress, anxiety, and depression in this demographic.

## CONCLUSIONS

The evidence has demonstrated that interventions aimed at promoting and reinforcing general self-efficacy beliefs in higher education students are effective. These interventions should incorporate content related to positive mental health, utilize psychoeducation strategies, span a duration of eight to twelve weeks, and integrate homework assignments. Nevertheless, the heterogeneity among studies in terms of objectives, content, strategies, duration, and sample size poses challenges when drawing conclusions.
